# Brain-to-text: decoding spoken phrases from phone representations in the brain

**DOI:** 10.3389/fnins.2015.00217

**Published:** 2015-06-12

**Authors:** Christian Herff, Dominic Heger, Adriana de Pesters, Dominic Telaar, Peter Brunner, Gerwin Schalk, Tanja Schultz

**Affiliations:** ^1^Cognitive Systems Lab, Institute for Anthropomatics and Robotics, Karlsruhe Institute of TechnologyKarlsruhe, Germany; ^2^New York State Department of Health, National Center for Adaptive Neurotechnologies, Wadsworth CenterAlbany, NY, USA; ^3^Department of Biomedical Sciences, State University of New York at AlbanyAlbany, NY, USA; ^4^Department of Neurology, Albany Medical CollegeAlbany, NY, USA

**Keywords:** electrocorticography, ECoG, speech production, automatic speech recognition, brain-computer interface, speech decoding, pattern recognition, broadband gamma

## Abstract

It has long been speculated whether communication between humans and machines based on natural speech related cortical activity is possible. Over the past decade, studies have suggested that it is feasible to recognize isolated aspects of speech from neural signals, such as auditory features, phones or one of a few isolated words. However, until now it remained an unsolved challenge to decode continuously spoken speech from the neural substrate associated with speech and language processing. Here, we show for the first time that continuously spoken speech can be decoded into the expressed words from intracranial electrocorticographic (ECoG) recordings.Specifically, we implemented a system, which we call *Brain-To-Text* that models single phones, employs techniques from automatic speech recognition (ASR), and thereby transforms brain activity while speaking into the corresponding textual representation. Our results demonstrate that our system can achieve word error rates as low as 25% and phone error rates below 50%. Additionally, our approach contributes to the current understanding of the neural basis of continuous speech production by identifying those cortical regions that hold substantial information about individual phones. In conclusion, the Brain-To-Text system described in this paper represents an important step toward human-machine communication based on imagined speech.

## 1. Introduction

Communication with computers or humans by thought alone, is a fascinating concept and has long been a goal of the brain-computer interface (BCI) community (Wolpaw et al., 2002). Traditional BCIs use motor imagery (McFarland et al., 2000) to control a cursor or to choose between a selected number of options. Others use event-related potentials (ERPs) (Farwell and Donchin, 1988) or steady-state evoked potentials (Sutter, 1992) to spell out texts. These interfaces have made remarkable progress in the last years, but are still relatively slow and unintuitive. The possibility of using covert speech, i.e., imagined continuous speech processes recorded from the brain for human-computer communication may improve BCI communication speed and also increase their usability. Numerous members of the scientific community, including linguists, speech processing technologists, and computational neuroscientists have studied the basic principles of speech and analyzed its fundamental building blocks. However, the high complexity and agile dynamics in the brain make it challenging to investigate speech production with traditional neuroimaging techniques. Thus, previous work has mostly focused on isolated aspects of speech in the brain.

Several recent studies have begun to take advantage of the high spatial resolution, high temporal resolution and high signal-to-noise ratio of signals recorded directly from the brain [electrocorticography (ECoG)]. Several studies used ECoG to investigate the temporal and spatial dynamics of speech perception (Canolty et al., 2007; Kubanek et al., 2013). Other studies highlighted the differences between receptive and expressive speech areas (Towle et al., 2008; Fukuda et al., 2010). Further insights into the isolated repetition of phones and words has been provided in Leuthardt et al. (2011b); Pei et al. (2011b). Pasley et al. (2012) showed that auditory features of perceived speech could be reconstructed from brain signals. In a study with a completely paralyzed subject, Guenther et al. (2009) showed that brain signals from speech-related regions could be used to synthesize vowel formants. Following up on these results, Martin et al. (2014) decoded spectrotemporal features of overt and covert speech from ECoG recordings. Evidence for a neural representation of phones and phonetic features during speech perception was provided in Chang et al. (2010) and Mesgarani et al. (2014), but these studies did not investigate continuous speech production. Other studies investigated the dynamics of the general speech production process (Crone et al., 2001a,b). A large number of studies have classified isolated aspects of speech processes for communication with or control of computers. Deng et al. (2010) decoded three different rhythms of imagined syllables. Neural activity during the production of isolated phones was used to control a one-dimensional cursor accurately (Leuthardt et al., 2011a). Formisano et al. (2008) decoded isolated phones using functional magnetic resonance imaging (fMRI). Vowels and consonants were successfully discriminated in limited pairings in Pei et al. (2011a). Blakely et al. (2008) showed robust classification of four different phonemes. Other ECoG studies classified syllables (Bouchard and Chang, 2014) or a limited set of words (Kellis et al., 2010). Extending this idea, the imagined production of isolated phones was classified in Brumberg et al. (2011). Recently, Mugler et al. (2014b) demonstrated the classification of a full set of phones within manually segmented boundaries during isolated word production.

To make use of these promising results for BCIs based on continuous speech processes, the analysis and decoding of isolated aspects of speech production has to be extended to continuous and fluent speech processes. While relying on isolated phones or words for communication with interfaces would improve current BCIs drastically, communication would still not be as natural and intuitive as continuous speech. Furthermore, to process the content of the spoken phrases, a textual representation has to be extracted instead of a reconstruction of acoustic features. In our present study, we address these issues by analyzing and decoding brain signals during continuously produced overt speech. This enables us to reconstruct continuous speech into a sequence of words in textual form, which is a necessary step toward human-computer communication using the full repertoire of imagined speech. We refer to our procedure that implements this process as *Brain-to-Text*. Brain-to-Text implements and combines understanding from neuroscience and neurophysiology (suggesting the locations and brain signal features that should be utilized), linguistics (phone and language model concepts), and statistical signal processing and machine learning. Our results suggest that the brain encodes a repertoire of phonetic representations that can be decoded continuously during speech production. At the same time, the neural pathways represented within our model offer a glimpse into the complex dynamics of the brain's fundamental building blocks during speech production.

## 2. Materials and methods

### 2.1. Subjects

**Figure 1:**
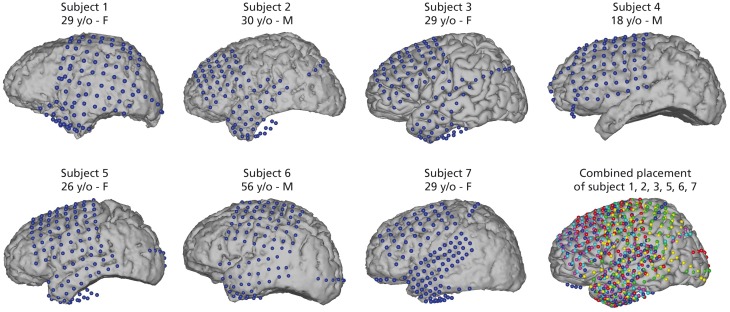
**Electrode positions for all seven subjects**. Captions include age [years old (y/o)] and sex of subjects. Electrode locations were identified in a post-operative CT and co-registered to preoperative MRI. Electrodes for subject 3 are on an average Talairach brain. Combined electrode placement in joint Talairach space for comparison of all subjects. Participant 1 (yellow), subject 2 (magenta), subject 3 (cyan), subject 5 (red), subject 6 (green), and subject 7 (blue). Participant 4 was excluded from joint analysis as the data did not yield sufficient activations related to speech activity (see Section 2.4).

Seven epileptic patients at Albany Medical Center (Albany, New York, USA) participated in this study. All subjects gave informed consent to participate in the study, which was approved by the Institutional Review Board of Albany Medical College and the Human Research Protections Office of the US Army Medical Research and Materiel Command. Relevant patient information is given in Figure 1.

### 2.2. Electrode placement

Electrode placement was solely based on clinical needs of the patients. All subjects had electrodes implanted on the left hemisphere and covered relevant areas of the frontal and temporal lobes. Electrode grids (Ad-Tech Medical Corp., Racine, WI; PMT Corporation, Chanhassen, MN) were composed of platinum-iridium electrodes (4 mm in diameter, 2.3 mm exposed) embedded in silicon with an inter-electrode distance of 0.6-1 cm. Electrode positions were registered in a post-operative CT scan and co-registered with a pre-operative MRI scan. Figure 1 shows electrode positions of all 7 subjects and the combined electrode positions. To compare average activation patterns across subjects, we co-registered all electrode positions in common Talairach space. We rendered activation maps using the NeuralAct software package (Kubanek and Schalk, 2014).

### 2.3. Experiment

We recorded brain activity during speech production of seven subjects using electrocorticographic (ECoG) grids that had been implanted as part of presurgical producedures preparatory to epilepsy surgery. ECoG provides electrical potentials measured directly on the brain surface at a high spatial and temporal resolution, unfiltered by skull and scalp. ECoG signals were recorded by BCI2000 (Schalk et al., 2004) using eight 16-channel g.USBamp biosignal amplifiers (g.tec, Graz, Austria). In addition to the electrical brain activity measurements, we recorded the acoustic waveform of the subjects' speech. Participant's voice data was recorded with a dynamic microphone (Samson R21s) and digitized using a dedicated g.USBamp in sync with the ECoG signals. The ECoG and acoustic signals were digitized at a sampling rate of 9600 Hz.

During the experiment, text excerpts from historical political speeches (i.e., Gettysburg Address, Roy and Basler, 1955), JFK's Inaugural Address (Kennedy, 1989), a childrens' story (Crane et al., 1867) or *Charmed* fan-fiction (Unknown, 2009) were displayed on a screen in about 1 m distance from the subject. The texts scrolled across the screen from right to left at a constant rate. This rate was adjusted to be comfortable for the subject prior to the recordings (rate of scrolling text: 42–76 words/min). During this procedure, subjects were familiarized with the task.

Each subject was instructed to read the text aloud as it appeared on the screen. A session was repeated 2–3 times depending on the mental and physical condition of the subjects. Table 1 summarizes data recording details for every session. Since the amount of data of the individual sessions of subject 2 is very small, we combined all three sessions of this subject in the analysis.

**Table 1:** **Data recording details for every session**.

**Participant**	**Session**	**Text**	**Number of phrases**	**Total recording length (s)**
1	1	Gettysburg address	36	279.87
	2	JFK inaugural	38	326.90
2	1	Humpty dumpty	21	129.87
	2	Humpty dumpty	21	129.07
	3	Humpty dumpty	21	126.37
3	1	Charmed fan-fiction	42	310.27
	2	Charmed fan-fiction	40	310.93
	3	Charmed fan-fiction	41	307.50
4	1	Gettysburg address	38	299.67
	2	Gettysburg address	38	311.97
5	1	JFK inaugural	49	341.77
	2	Gettysburg address	39	222.57
6	1	Gettysburg address	38	302.83
7	1	JFK inaugural	48	590.10
	2	Gettysburg address	38	391.43

**Figure 2:**
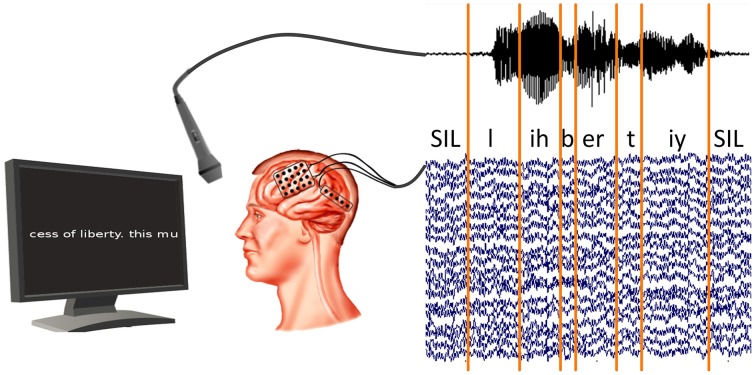
**Synchronized recording of ECoG and acoustic data**. Acoustic data are labeled using our in-house decoder BioKIT, i.e., the acoustic data samples are assigned to corresponding phones. These phone labels are then imposed on the neural data.

We cut the read-out texts of all subjects into 21–49 phrases, depending on the session length, along pauses in the audio recording. The audio recordings were phone-labeled using our in-house speech recognition toolkit BioKIT Telaar et al., 2014 (see Section 2.5). Because the audio and ECoG data were recorded in synchronization (see Figure 2), this procedure allowed us to identify the ECoG signals that were produced at the time of any given phones. Figure 2 shows the experimental setup and the phone labeling.

### 2.4. Data pre-selection

In an initial data pre-selection, we tested whether speech activity segments could be distinguished from those with no speech activity in ECoG data. For this purpose, we fitted a multivariate normal distribution to all feature vectors (see Section 2.6 for a description of the feature extraction) containing speech activity derived from the acoustic data and one to feature vectors when the subject was not speaking. We then determined whether these models could be used to classify general speech activity above chance level, applying a leave-one-phrase-out validation.

Based on this analysis, both sessions of subject 4 and session 2 of subject 5 were rejected, as they did not show speech related activations that could be classified significantly better than chance (*t*-test, *p* > 0.05). To compare against random activations without speech production, we employed the same randomization approach as described in Section 2.11.

### 2.5. Phone labeling

Phone labels of the acoustic recordings were created in a three-step process using an English automatic speech recognition (ASR) system trained on broadcast news. First, we calculated a Viterbi forced alignment (Huang et al., 2001), which is the most likely sequence of phones for the acoustic data samples given the words in the transcribed text and the acoustic models of the ASR system. In a second step, we adapted the Gaussian mixture model (GMM)-based acoustic models using maximum likelihood linear regression (MLLR) (Gales, 1998). This adaptation was performed separately for each session to obtain session-dependent acoustic models specialized to the signal and speaker characteristics, which is known to increase ASR performance. We estimated a MLLR transformation from the phone sequence computed in step one and used only those segments which had a high confidence score that the segment was emitted by the model attributed to them. Third, we repeated the Viterbi forced alignment using each session's adapted acoustic models yielding the final phone alignments. The phone labels calculated on the acoustic data are then imposed on the ECoG data.

Due to the very limited amount of training data for the neural models, we reduced the amount of distinct phone types and grouped similar phones together for the ECoG models. The grouping was based on phonetic features of the phones. See Table 2 for the grouping of phones.

**Table 2:** **Grouping of phones**.

**Grouped phone**	**IPA phones**
aa	ɑ æ ʌ
b	ɓ
ch	ʈ ʃʃ ʒ
eh	ɛ ɝ eɪ
f	f
hh	h
ih	i ɪ
jh	dʒ g j
k	k
l	ɫ
m	m
n	n ŋ
ow	o ℧ ɔ
p	p
r	r
s	s z ð θ
t	t d
uw	u ℧
v	v
w	w
**Diphtongs**	
ow ih	ɔɪ
aa ih	aɪ
aa ow	a℧

*English phones are based on the International Phonetic Alphabet (IPA)*.

### 2.6. Feature extraction

We segmented the neural signal data continuously into 50 ms intervals with an overlap of 25 ms, which enabled us to capture the fast cortical processes underlying phones, while being long enough to extract broadband (70–170 Hz) gamma activity reliably. Each of the 50 ms intervals was labeled with the corresponding phone obtained from the audio phone labeling. We extracted broadband-gamma activations as they are known to be highly task-related for motor tasks (Miller et al., 2007), music perception (Potes et al., 2012), auditory processes (Pei et al., 2011b; Pasley et al., 2012) and word repetition (Leuthardt et al., 2011b). Broadband-gamma features were extracted from the ECoG electrical potentials as follows: linear trends in the raw signals were removed from each channel. The signals were down-sampled from 9600 to 600 Hz sampling rate. Channels strongly affected by noise were identified and excluded from further processing. Specifically, we calculated the energy in the frequency band 58–62 Hz (line noise) and removed channels with more noise energy than two interquartile ranges above the third quartile of the energy of all channels in the data set. This way, an average of 7.0 (std 6.5) channels were removed per subject.

The remaining channels were re-referenced to a common average (i.e., CAR filtering). Elliptic IIR low-pass and high-pass filters were applied to represent broadband gamma activity in the signals. An elliptic IIR notch filter (118–122 Hz, filter order 13) was applied to attenuate the first harmonic of 60 Hz line noise, which is within the broadband gamma frequency range.

Resulting 50 ms intervals are denoted as *X*_*i,c*_(*t*) and consist of *n* samples (*t* ∈ [1, …, *n*]). For each interval *i* and channel *c*, the signal energy *E*_*i,c*_ was calculated and the logarithm was applied to make the distribution of the energy features approximately Gaussian: $E_{i,c}=log(\frac{1}{n}\sum_{t=1}^{n}X_{i,c}{\left(t\right)}^{2})$. The logarithmic broadband gamma power of all channels were concatenated into one feature vector *E*_*i*_ = [*E*_*i*,1_, …, *E*_*i,d*_]. To integrate context information and temporal dynamics of the neural activity for each interval, we included neighboring intervals up to 200 ms prior to and after the current interval, similar context sizes have been found relevant in speech perception studies Sahin et al., 2009. Therefore, each feature vector was stacked with four feature vectors in the past and four feature vectors in the future. Stacked feature vectors *F*_*i*_ = [*E*_*i*−4_, …, *E*_*i*_, …, *E*_*i*+4_]⊤ were extracted every 25 ms over the course of the recording sessions and the fitting phone label (ground truth from acoustic phone labeling) was associated.

### 2.7. Identification of discriminability

The high temporal and spatial resolution of ECoG recordings allowed us to trace the temporal dynamics of speech production through the areas in the brain relevant for continuous natural speech production. To investigate such cortical regions of high relevance, we calculated the mean symmetrized Kullback-Leibler divergence (KL-div) among the phone models for each electrode position and at every time interval.

The Kullback-Leibler divergence (KL-div) is a measure of the difference between two distributions *P* and *Q*. It can be interpreted as the amount of discriminability between the neural activity models in bits. It is non-symmetric and does not satisfy the triangle inequality. The KL-div can be interpreted as the amount of extra bits needed to code samples from *P* when using *Q* to estimate *P*. When both distributions *P* and *Q* are normal distributions with means μ_0_ and μ_1_ and covariances Σ_0_ and Σ_1_, respectively, the KL-div can be easily calculated as

(1) $$\begin{array}{l} D_{KL}(N_{0}||N_{1})=\frac{1}{2}(tr(\Sigma_{1}^{-1}\Sigma_{0})+{\left(\mu_{1}-\mu_{0}\right)}^{T}\Sigma_{1}^{-1}(\mu_{1}-\mu_{0})\\ \;-d-log_{2}(\frac{det(\Sigma_{0})}{det(\Sigma_{1})})) \end{array}$$

with *d* being the dimensionality of the distributions. The closed-form of the KL-div enables us to calculate the difference between two phone models. To estimate the discriminability of a feature *E*_*i,c*_ (log broadband gamma power of a particular channel and time interval) for the classification of phones, we calculate the mean KL-div between all pairs of phones for this particular feature. The mean between all divergences symmetrizes the KL-div and yields one number in bits as the estimation of the discriminability of this particular feature *E*_*i,c*_.

### 2.8. Feature selection

We selected features with the largest average distance between phone models based on the mean KL-div (cf. previous section) in the training data during each run of the leave-one-phrase-out validation. The number of features selected was automatically determined based on the distribution of KL-div for this specific run as follows: We normalized the mean KL-div values *d*_*k*_ for every feature *k* by their average (${\hat{d}}_{k}=\frac{d_{k}}{\sum_{k}d_{k}}$). Then, we sorted the values in descending order and selected features with large normalized mean KL-div until the sorted sequence did not decline more than a threshold *t* = −0.05: arg max_*l*_ *sort*($\hat{d}$_*k*_)_*l*_ − −*sort*($\hat{d}$_*k*_)_*l*+1_ < *t*. The threshold value *t* = −0.05 corresponds to a very low decline in KL-div and thus reflected the point after which little additional information was present. This way, only the *l* most relevant features are selected to limit the feature space.

Note that features are selected solely based on the Kullback-Leibler divergence in the training data and do not include any prior assumptions on the suitability of specific regions for phone discrimination. We further reduced the feature space dimensionality by linear discriminant analysis (LDA) (Haeb-Umbach and Ney, 1992) using the phone labels on the training data.

### 2.9. ECoG phone model training

Each phone was modeled in the extracted feature space by a normal distribution. Thus, models characterized the mean contribution and variance of the neural activity measured at each electrode. We represented the stacked cortical activity feature vectors *F*_*i*_ of each phone *j* by a model λ_*j*_ as a multivariate Gaussian probability density function *p*(*F*_*i*_|λ_*j*_) ~ 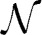
(μ_*j*_,Σ_*j*_) determined by the mean feature vectors μ_*j*_ and their diagonal variance matrix Σ_*j*_ calculated from training data. Gaussian models were chosen as they represent the underlying feature distribution suitably well. Furthermore, Gaussian models can be robustly calculated from a small amount of data, they are computationally very efficient and allow a closed form calculation of the Kullback-Leibler-Divergence.

### 2.10. Decoding approach

Following a common idea of modern speech recognition technology (Rabiner, 1989; Schultz and Kirchhoff, 2006), we combined the information about the observed neural activity with statistical language information during the decoding process by Bayesian updating (Rabiner, 1989). Simplified, the process can be understood (Gales and Young, 2008) as finding the sequence of words *W* = *w*_1_ … *w*_*L*_ which is most likely given the observed ECoG feature segments *X* = *F*_1_ … *F*_*T*_. This probability *P*(*W*|*X*) can be transformed using Bayes' rule:

(2) $$\hat{W}=\underset{\;W}{\arg\max}\{P(W|X)\}=\underset{\;W}{\arg\max}\{p(X|W)P(W)\}$$

Here, the likelihood *p*(*X*|*W*) is given by the ECoG phone models and *P*(*W*) is calculated using a language model. The likelihood of ECoG phone models *p*(*X*|*W*) given a word *W* is calculated by concatenating ECoG phone models to form words as defined in a pronunciation dictionary. Specifically, we employed a pronunciation dictionary containing the mapping of phone sequences to words, for example, describing that the word “liberty” comprises of the phone sequence “*/l/ /ih/ /b/ /er/ /t/ /iy/.”* We constructed a minimized and determinized search graph consisting of the phone sequences for each recognizable word. To capture important syntactic and semantic information of language, we used a statistical language model (Jelinek, 1997; Stolcke, 2002) that predicts the next word given the preceding words. In N-gram language modeling, this is done by calculating probabilities of single words and probabilities for predicting words given the *n* − 1 previous words. Probabilities for single word occurrence (*n* = 1) are called uni-grams. Probabilities for the co-occurrence of two words (*n* = 2) are called bi-grams. For the *Brain-to-Text* system, we estimate bi-grams on the texts read by the subjects. It is important to note that even though this results in very specialized models, the correctness of our results is still assured, as the same language models are utilized for both the real as well as for the control analyses.

Finally, the decoding of spoken phrases from neural data *X* is performed by finding the word sequence *Ŵ* in the search graph that has the highest likelihood for producing the neural data with respect to the ECoG phone models and language information given by pronunciation dictionary and language model.

**Figure 3:**
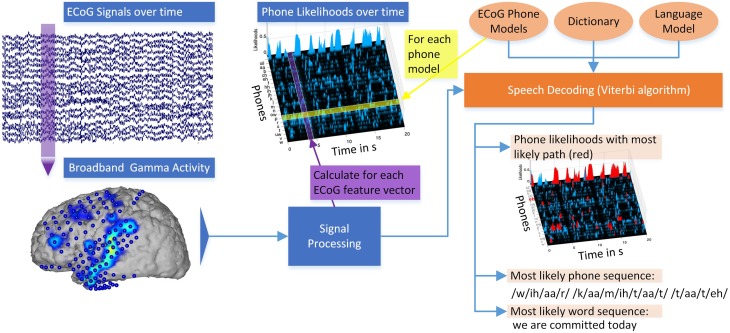
**Overview of the** ***Brain-to-Text*** **system:** ECoG broadband gamma activities (50 ms segments) for every electrode are recorded. Stacked broadband gamma features are calculated (Signal processing). Phone likelihoods over time can be calculated by evaluating all Gaussian ECoG phone models for every segment of ECoG features. Using ECoG phone models, a dictionary and an n-gram language model, phrases are decoded using the Viterbi algorithm. The most likely word sequence and corresponding phone sequence are calculated and the phone likelihoods over time can be displayed. Red marked areas in the phone likelihoods show most likely phone path. See also Supplementary Video.

Figure 3 illustrates the different steps of decoding continuously spoken phrases from neural data. *ECoG signals over time* are recorded at every electrode and divided into 50 ms segments. For each 50 ms interval of recorded *broadband gamma activity*, stacked feature vectors are calculated (*Signal processing*). For each *ECoG phone model* calculated on the training data, the likelihood that this model emitted a segment of ECoG features can be calculated, resulting in *phone likelihoods over time*. Combining these Gaussian *ECoG phone models* with language information in the form of a *dictionary* and an n-gram *language model*, the *Viterbi* algorithm calculates the *most likely word sequence* and corresponding *phone sequence*. To visualize the decoding path, the *most likely phone sequence* can be shown in the *phone likelihoods over time* (red marked areas). The system outputs the decoded word sequence. Overall, the system produces a textual representation from the measured brain activity (see also Supplementary Video).

### 2.11. Evaluation

For the evaluation of our *Brain-to-Text* system, we trained neural phone models using all but one phrase of a recording session and decoded the remaining phrase. This evaluation process was repeated for each phrase in the session. Through this leave-one-phrase-out validation, we make sure that all feature selection, dimensionality reduction and training steps are only performed on the training data while the test data remains completely unseen. For comparison, we performed the decoding with randomized phone models. This is a baseline that quantifies how well the language model and dictionary decode phrases without any neural information. To obtain an estimate for chance levels in our approach, we shifted the training data by half its length in each iteration of the leave-one-phrase-out validation while the corresponding labels remained unchanged. This way, the data for the random comparison models still have the typical properties of ECoG broadband gamma activity, but do not correspond to the underlying labels. Furthermore, as the labels are not changed, prior probabilities remain the same for the random and the actual model case. As the shifting point is different for all iterations of the specific session, we get an estimate of the chance level performance for every phrase. The mean over all these results thus allows a robust estimation of the true chance level (randomization test).

It is also important to bear in mind that *Brain-to-Text* is still at a disadvantage compared to traditional speech recognition systems as our data contained only several minutes of ECoG signals for each subject. This limited model complexity compared to traditional speech recognition systems, which are usually trained on thousands of hours of acoustic data and billions of words for language model training.

We evaluated the performance of our *Brain-to-Text* system with different dictionary sizes. For this purpose, we created new dictionaries for every test phrase including the words that were actually spoken plus a set of randomized set of words from the full dictionary. Created dictionaries were the same for *Brain-To-Text* and randomized models to ensure that the words chosen had no influence on the comparison between models. The language model was limited to the words in the dictionary accordingly. This approach allowed us to perpetually increase the dictionary size.

## 3. Results

### 3.1. Regions of discriminability

**Figure 4:**
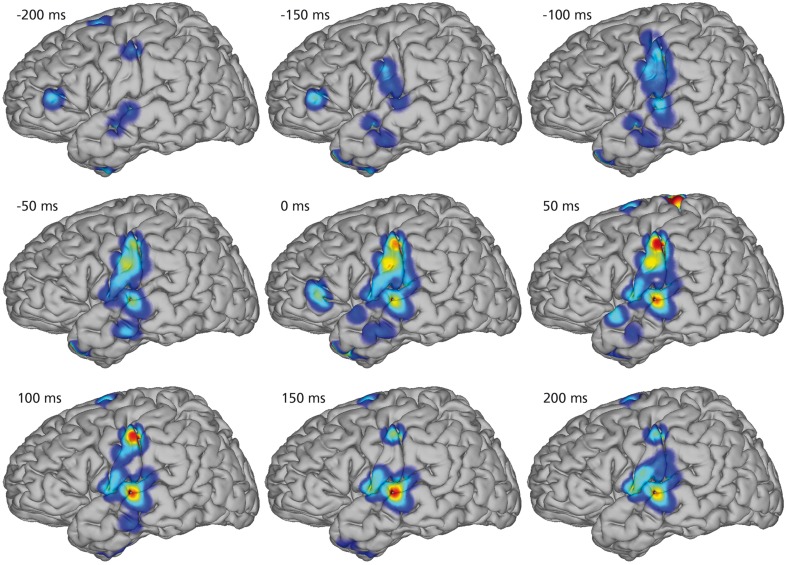
**Mean Kullback-Leibler Divergences between models for every electrode position of every subject**. Combined electrode montage of all subjects except subject 4 in common Talairach space. Heat maps on rendered average brain shows regions of high discriminability (red). All shown discriminability exceeds chance level (larger than 99% of randomized discriminabilities). The temporal course of regions with high discriminability between phone models shows early differences in diverse areas up to 200 ms before the actual phone production. Phone models show high discriminability in sensorimotor cortex 50 ms before production and yield different models in auditory regions of the superior temporal gyrus 100 ms after production.

Figure 4 illustrates the spatio-temporal dynamics of the mean KL-div between the phone models on a joint brain surface (Talairach model, Talairach and Tournoux, 1988) for nine temporal intervals with co-registered electrodes of all subjects. KL-div values plotted in Figure 4 exceed 99% of the KL-div values with a randomized phone-alignment (data shifted by half its length while the labels remain the same).

Starting 200 ms before the actual phone production, we see high KL-div values in diverse areas including Broca's area, which is generally associated with speech planning (Sahin et al., 2009). 150 ms prior to the phone production, Broca's area still has high KL-div scores, but additionally sensorimotor areas and regions in the superior temporal gyrus associated with auditory and language function show increasing discriminability. Subsequently, activations in Broca's area vanish and motor area discriminability increases until peaking at the interval between 0 and 50 ms (which corresponds to the average length of phones). Discriminability increases in auditory regions until approximately 150 ms after phone production.

### 3.2. Decoding results

**Figure 5:**
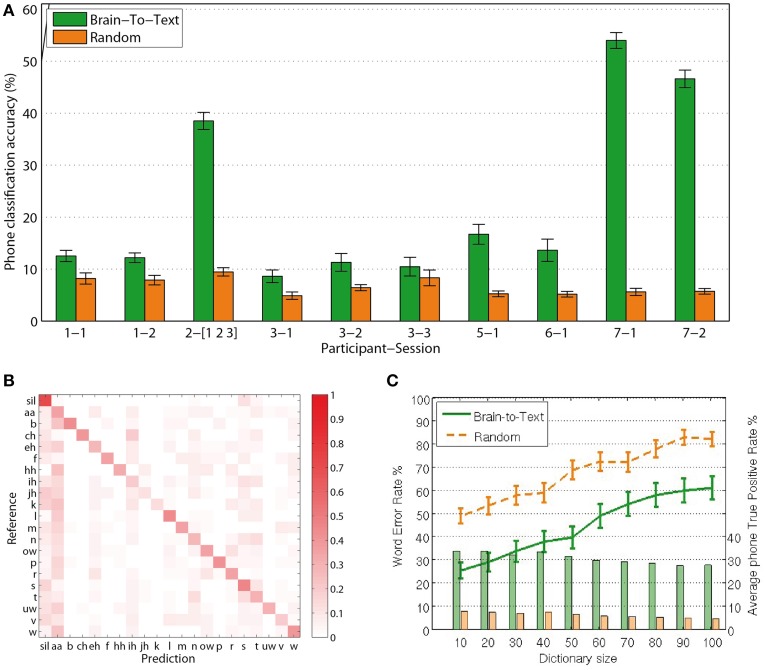
**Results: (A)** Frame-wise accuracy for all sessions. All sessions of all subjects show significantly higher true positive rates for *Brain-To-Text* (green bars) than for the randomized models (orange bars). **(B)** Confusion matrix for subject 7, session 1. The clearly visible diagonal indicates that all phones are decoded reliably. **(C)** Word Error Rates depending on dictionary size (lines). Word error rates for *Brain-To-Text* (green line) are lower than the randomized models for all dictionary sizes. Average true-positive rates across phones depending on dictionary size (bars) for subject 7, session 1. Phone true positive rates remain relatively stable for all dictionary sizes and are always much higher for *Brain-To-Text* than for the randomized models.

For each phrase to be decoded, the most likely phone-path can be efficiently calculated using Viterbi decoding (Rabiner, 1989). Comparing the extracted phone labels for each feature vector with the baseline labels from the audio alignment, we calculate single-frame accuracies for the decoding of phones from continuous speech production. Reducing the size of the dictionary to 10 words, including those that are to be evaluated, *Brain-to-Text* yielded significantly higher accuracies (two-sided *t*-test, *p* < 0.05 for all sessions) for single phone decoding in all sessions compared to random models. Figure 5A shows average phone recognition accuracies (green) and average random recognition accuracies (orange) for each session. The best session resulted in average accuracies above 50% for the correct classification of 20 phones plus Silence. While all sessions resulted in significantly higher accuracies than random models, the results of subject 2 and subject 7 clearly outperform those of all other subjects. The outstanding performance of subject 7 might be explained by the high-density grid on the superior temporal gyrus. We further investigate the results of subject 7, session 1 (results for all other subjects and sessions can be found in the Supplementary Material) by investigating the confusion matrix (Figure 5B) that shows which phones in the reference corresponded to which phones in the predicted phrase. The clearly visible diagonal in this confusion matrix illustrates that our approach reliably decodes the complete set of phones.

In *Brain-to-Text*, we decode entire word sequences of each test phrase. Even with a small dictionary size, a large number of different phrases can be produced, as the number of words may vary and words can be arbitrarily combined. Therefore, we utilize the Word Error Rate (WER) to measure the quality of a decoded phrase. The word error rate (WER) between a predicted phrase and the corresponding reference phrase consists of the number of editing steps in terms of substitutions, deletions and insertions of words necessary to produce the predicted phrase from the reference, divided by the amount of words in the reference.

Figure 5C shows the average WER depending on dictionary size (green line). For all dictionary sizes, the performance is significantly better than randomized results (orange line). Significance was analyzed using paired *t*-tests between the Word Error Rates of *Brain-To-Text* and the randomized models (*p* < 0.001, one-sided paired *t*-test). With 10 words in the dictionary, 75% of all words are recognized correctly. The approach scales well for increasing dictionary sizes. Average phone true positive rates remain rather stable even when dictionary sizes increase (bars in Figure 5C).

## 4. Discussion

### 4.1. ECoG phone models

Gaussian models as a generative statistical representation for log-transformed broadband gamma power have been found well-suited for the observed cortical activity (e.g., Gasser et al., 1982; Crone et al., 2001b). These models facilitate the analysis of the spatial and temporal characteristics of each phone model within its 450 ms context. Note that the modeling of phones does not contradict recent findings of articulatory features in neural recordings during speech perception (Pulvermüller et al., 2006; Mesgarani et al., 2014) and production (Bouchard et al., 2013; Lotte et al., 2015), since multiple representations of the same acoustic phenomenon are likely.

Note that only one context-independent model is trained for each phone, i.e., without consideration of preceding or succeeding phones due to the limited amount of data, even though effects of context have been shown in neural data (Mugler et al., 2014a). While context dependent modeling is very common in acoustic speech recognition (Lee, 1990) and known to significantly improve recognition performance, it requires substantially more training data than available in our ECoG setting.

### 4.2. Regions of discriminability

In our approach, the phone representation through Gaussian models allows for detailed analysis of cortical regions, which have high discriminability among the different phones over time. The cortical locations identified using the KL-div criterion are in agreement with those that have been identified during speech production and perception in isolated phoneme or word experiments (Canolty et al., 2007; Leuthardt et al., 2011a). These findings extend the state-of-the-art by showing for the first time the dynamics for single phone discriminability and decoding during continuous speech production.

As our experiments demand overt speech production from prompted texts, it is evident that multiple processes are present in the recorded neural data, including speech production, motor actions, auditory processing, and language understanding. By demonstrating that phones can be discriminated from each other, we show that such a phone-based representation is indeed a viable form of modeling cortical activity of continuous speech in this mixture of activation patterns.

### 4.3. Decoding results

The reported phone decoding accuracies are significantly higher for *Brain-to-Text* than for randomized models in all subjects, which shows that continuous speech production can be modeled based on phone representations. The clearly visible diagonal in the confusion matrix Figure 5B emphasizes that the decoding performance is based on a reliable detection of all phones and not only on a selected subset.

Different conditions, such as varying task performance of the subjects, and different positions and densities of the electrode grids, yielded highly variable decoding performances for the different subjects, however the low WER (see Supplementary Material) and phone true positive rates for subject 1,2, and 7 imply the potential of *Brain-to-Text* for brain-computer interfaces.

### 4.4. Conclusion

Decoding overt speech production is a necessary first step toward human-computer interaction through imagined speech processes. Our results show that with a limited set of words in the dictionary, *Brain-to-Text* reconstructs spoken phrases from neural data. The computational phone models in combination with language information make it possible to reconstruct words in unseen spoken utterances solely based on neural signals (see Supplementary Video). Despite the fact that the evaluations in this article have been performed offline, all processing steps of *Brain-to-Text* and the decoding approach are well suited for eventual real-time online application on desktop computers. The approach introduced here may have important implications for the design of novel brain-computer interfaces, because it may eventually allow people to communicate solely based on brain signals associated with natural language function and with scalable vocabularies.

## Funding

This work was supported by the NIH (EB00856, EB006356, and EB018783), the US Army Research Office (W911NF-08-1-0216, W911NF-12-1-0109, W911NF-14-1-0440) and Fondazione Neuron, and received support by the International Excellence Fund of Karlsruhe Institute of Technology. We acknowledge support by Deutsche Forschungsgemeinschaft and Open Access Publishing Fund of Karlsruhe Institute of Technology.

### Conflict of interest statement

The authors declare that the research was conducted in the absence of any commercial or financial relationships that could be construed as a potential conflict of interest.
