# Supplementary material for: Brain-to-text: decoding spoken phrases from phone representations in the brain
Source: Front Neurosci. 2015 Jun 12;9:217. doi: 10.3389/fnins.2015.00217 (PMC4464168; doi:10.3389/fnins.2015.00217)
Supplement: Supplementary file 2 [file Image1.PDF]

Frontiers Neuroengineering

Research Article
28 January 2015

Supplementary Material: Article Title

Christian Herff[1*], Dominic Heger[1*], Adriana de Pesters[2,4], Dominic Telaar[1], Peter Brunner[2,3], Gerwin Schalk[2,3,4], Tanja Schultz[1]

[1] *Cognitive Systems Lab, Institute for Anthropomatics, Karlsruhe Institute of Technology, Karlsruhe, Germany*
[2] *National Center for Adaptive Neurotechnologies, Wadsworth Center, New York State Department of Health, Albany, NY, USA*
[3] *Department of Neurology, Albany Medical College, Albany, USA*
[4] *Department of Biomedical Science, State University of New York at Albany, Albany, NY, USA*

Correspondence*:
Christian Herff
Cognitive Systems Lab, Institute for Anthropomatics and Robotics, Karlsruhe Institute of Technology, Adenauerring 4, 76131 Karlsruhe, Germany, christian.herff@kit.edu

Dominic Heger
Cognitive Systems Lab, Institute for Anthropomatics and Robotics, Karlsruhe Institute of Technology, Adenauerring 4, 76131 Karlsruhe, Germany, dominic.heger@kit.edu
* These authors contributed equally to this work.

1 SUPPLEMENTARY DATA

The full results for all participants

2 SUPPLEMENTARY TABLES AND FIGURES

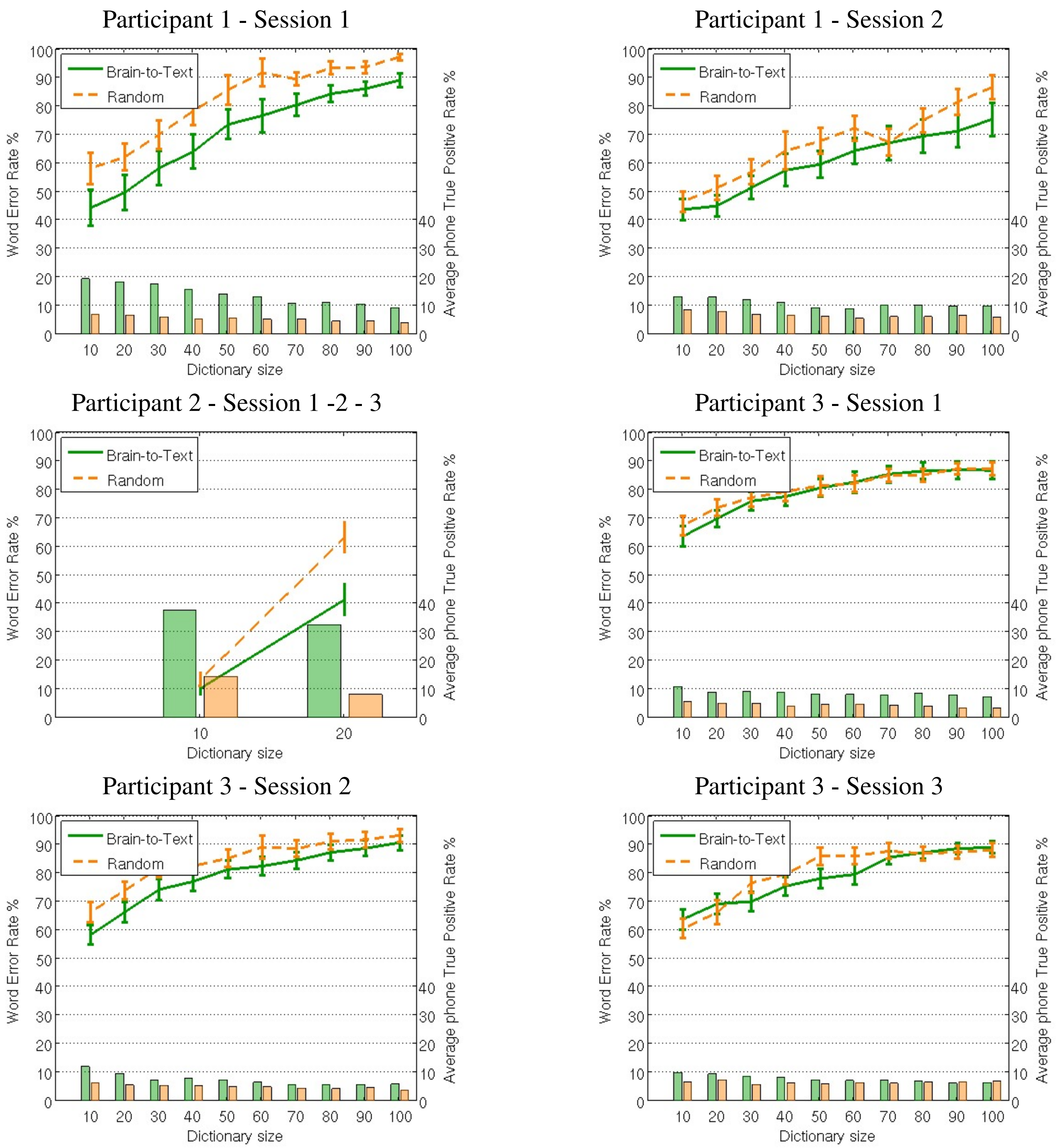

Figure 1. WER results and average phone true positive rate over dictionary size for all sessions and participants.

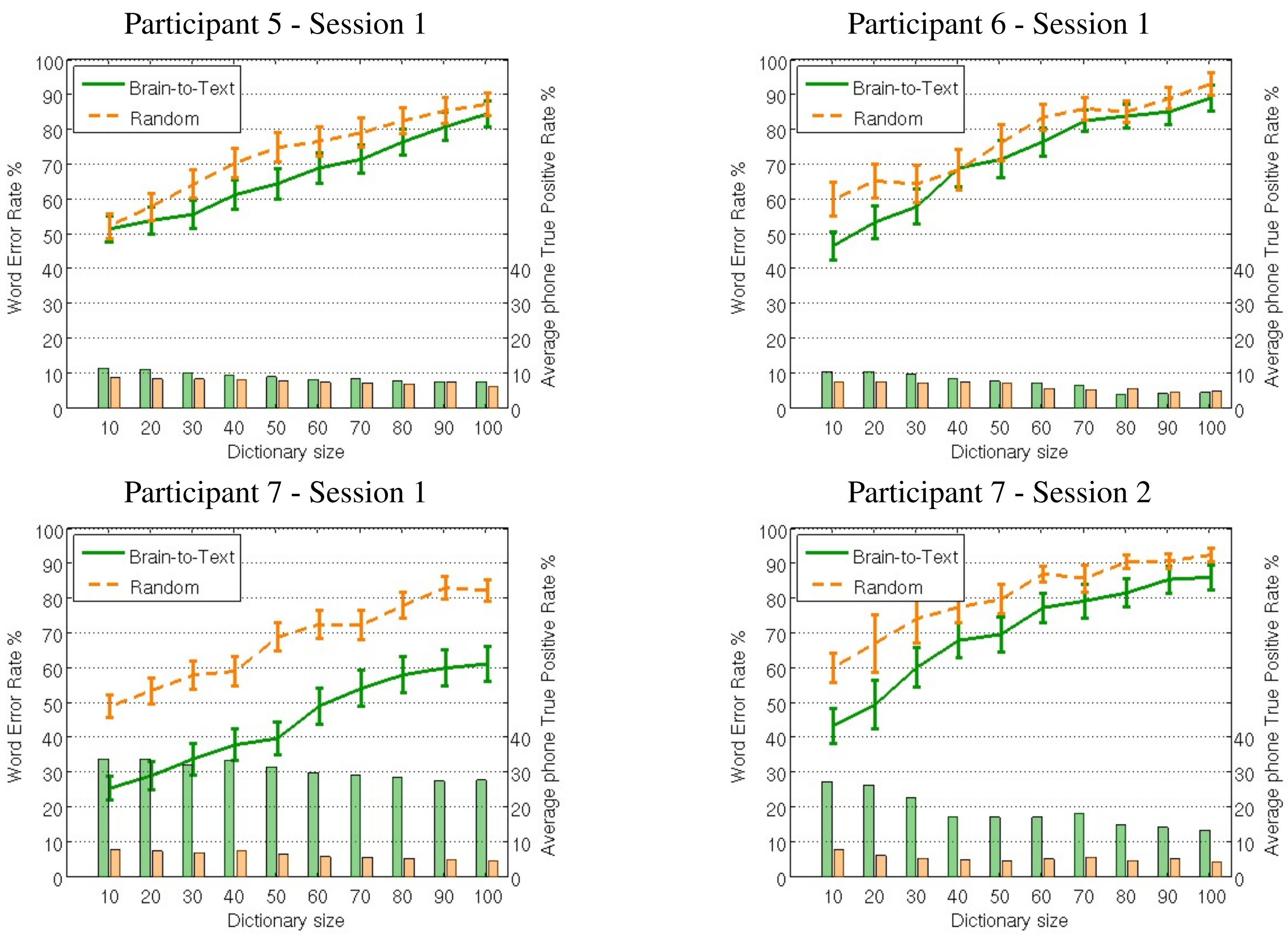

Figure 2. WER results and average phone true positive rate over dictionary size for all sessions and participants.
